# Reply to the Letter: “Uncovering the Missing Links: Autoimmunity and Infection in Pediatric Arterial Ischemic Stroke”

**DOI:** 10.1055/s-0046-1817047

**Published:** 2026-04-15

**Authors:** Muharrem Bostancı, Arzu Ekici, Cengiz Havalı

**Affiliations:** 1 University of Health Sciences, Bursa Yuksek Ihtisas Training and Research Hospital, Department of Pediatrics, Bursa, Turkey.; 2 Basaksehir Cam and Sakura City Hospital Istanbul, Turkey.

Dear Editor,

Thank you for your letter and for the opportunity to clarify our approach. We appreciate the reviewers' careful reading and agree that autoimmune and infectious etiologies are important considerations in pediatric ischemic stroke.

Major pediatric stroke reviews and clinical guidelines recommend investigating infectious and autoimmune causes as part of a comprehensive etiologic workup in children with AIS, because identifying a specific underlying cause affects prognosis and therapy (such as immunosuppression for CNS vasculitis, antimicrobial therapy for an infectious arteriopathy, or long-term anticoagulation for antiphospholipid syndrome). The Royal College of Paediatrics and Child Health (RCPCH) and widely-cited pediatric stroke reviews 1 2 emphasize the inclusion of infectious and inflammatory testing when indicated by history, imaging, or initial laboratory results.

Pediatric stroke guidelines recommend that autoimmune or infectious testing should be guided by history (recent infection, fever, rash, autoimmune symptoms), examination (signs of systemic inflammation, mucocutaneous findings), imaging (arteriopathy, vasculitic pattern), and basic labs (elevated erythrocyte sedimentation rate (ESR)/C-reactive protein (CRP), cytopenia, thrombocytosis); thus routine broad screening without clinical cues is not recommended. 1 2

Several studies 3 4 show that positivity for antinuclear antibodies (ANAs) may be present in up to 10 to 15% of healthy children, even in the absence of signs or symptoms of autoimmune disease. In such low pretest probability contexts, the positive predictive value for a true connective tissue disease is very low. Similarly, antiphospholipid antibodies have been documented transiently in asymptomatic children—such as after minor surgery or viral infections—without evolving into a clinical syndrome. Therefore, performing or interpreting serologic tests in the absence of clinical suspicion risks false-positive or irrelevant findings and may lead to misclassification. Hence, in pediatric stroke evaluation, serology should be reserved for cases with clinical, imaging or laboratory indicators suggesting inflammatory or thrombotic phenotypes.

The objective of our study was to delineate the presenting symptoms and etiological factors in pediatric patients with arterial ischemic stroke. Although it may not have been fully elaborated in the “Methods” section, anti-double-stranded DNA (anti-dsDNA) antibodies and ANAs were tested in all patients. Due to negative results and lack of additional supporting findings, no further investigation was conducted. However, it would be appropriate to look for lupus anticoagulants and anticardiolipin antibodies, especially in the adolescent age group. The iInvestigation of collagen tissue diseases is recommended in patients with multiple, widespread, small and medium-sized infarct lesions, especially those located in cortical and subcortical areas, involving more than one arterial region. 4

As the reviewers correctly noted, infections such as those by the *Herpes simplex* virus and varicella-zoster virus can contribute to ischemic events in children. However, none of the patients in our cohort exhibited clinical findings suggestive of these infections; therefore, routine serologic testing was not performed. Among the few patients for whom serologic assays were obtained, all results were negative; thus, they were not considered clinically meaningful or relevant to the study findings. We did not include these results in the manuscript because viral serologic tests carry a substantial risk of false-positive or misleading interpretations when they are not supported by compatible clinical history or examination findings. 5 Although PCR is more specific than serology, it does not always reflect true infection. False-negative PCR results may occur due to low viral load, suboptimal sample collection, or specimen handling issues, while false-positive results may arise from contamination or persistence of non-viable viral nucleic acids. Thus, PCR findings must be interpreted within the clinical context rather than used as universal screening in pediatric stroke. Vora et al., 6 for example, noted that the patient's sibling had developed a vesicular rash 2 to 3 months earlier, and the patient himself had experienced a mild rash. In this context, the patient's serologic evaluation showed positive varicella immunoglobulin G (IgG) and negative immunoglobulin G (IgM). In such a case, the serologic findings are considered meaningful only because they correlate with the clinical scenario (that is, household exposure and compatible rash). Without these clinical clues, an isolated positive varicella IgG result would not be sufficient to establish relevance or causality.

The data was collected for our study from 2016 to 2020, and the first confirmed case of severe acute respiratory syndrome coronavirus 2 (SARS-CoV-2) infection in Turkey was reported in March 2020. Only two patients in our cohort were admitted in 2020, and both had negative PCR results for SARS-CoV-2. For cases predating 2020, routine coronavirus serologic testing was not performed; therefore, no definitive information regarding prior coronavirus exposure is available for those patients.

In conclusion, autoimmune and infectious mechanisms were incorporated into our etiologic evaluation because both are recognized contributors to pediatric arterial ischemic stroke, particularly in cases in which clinical history, examination, or neuroimaging suggests an underlying inflammatory or immune-mediated process. Nevertheless, current pediatric stroke guidelines emphasize that extensive serologic and molecular testing should not be undertaken routinely in the absence of specific clinical indicators. This is particularly important because viral serologies—especially IgM assays—are susceptible to false-positive results, and PCR testing may yield false negatives or false positives depending on sampling technique, timing, and laboratory factors. For these reasons, a focused, hypothesis-driven diagnostic approach remains the most reliable and evidence-based strategy to identify infectious or autoimmune etiologies in children presenting with stroke.
